# Association between long Internet use during pregnancy and low birth weight: a retrospective cohort study

**DOI:** 10.1265/ehpm.24-00279

**Published:** 2024-12-12

**Authors:** Aya Sakakihara, Chiyori Haga, Aya Kinjo, Yoneatsu Osaki

**Affiliations:** 1 Department of Community Health Nursing, Faculty of Medicine, Shimane University, 89-1 Enya-cho, Izumo City, Shimane Prefecture 693-8501, Japan; 2 Graduate School of Medicine, Department of Community Nursing, Kagawa University, 1750-1 Ikenobe, Miki-cho, Kita-gun, Kagawa Prefecture 761-0793, Japan; 3 Division of Environmental and Preventive Medicine, Faculty of Medicine, Tottori University, 86 Nishi-cho, Yonago City, Tottori Prefecture 683-8503, Japan

**Keywords:** Low birth weight, Long internet use, Problematic internet use, Pregnancy period, Retrospective cohort study

## Abstract

**Background:**

Low birth weight (LBW) is an important public health issue that affects development and health over a long period. However, there has been no sufficient decrease in the prevalence of LBW, and it is important to identify preventable factors for LBW which remain to be clarified. The purpose of this study was to clarify the association between Internet use for many hours during pregnancy and LBW.

**Methods:**

The subjects were mothers who had submitted the pregnancy notification form in Matsue City between April 2016 and September 2017 and their children. The data provided by Matsue City authorities consisted of 2,465 records. We analyzed 2,089 records, excluding untraceable records, those with insufficient information, those on multiple pregnancy, and those on pregnant smokers. Logistic regression analysis was performed using LBW as a dependent variable, Internet use for many hours during pregnancy as an independent variable, and the child’s sex, mother’s age at the time of pregnancy, unmarried status on pregnancy, first childbirth, mother’s job during pregnancy, and weeks of pregnancy on the notification as covariates.

**Results:**

The results of analysis showed that Internet use for many hours during pregnancy accounted for 4.4%, and that LBW accounted for 7.2%. Internet use for many hours during pregnancy was associated with LBW (adjusted odds ratio = 2.16 (95%CI: 1.13–4.13)).

**Conclusions:**

This study suggested that there is an association between Internet use for many hours during pregnancy and LBW. It is necessary to provide appropriate support to pregnant women who use the Internet for many hours during pregnancy after confirming the presence or absence of risk factors for LBW.

**Supplementary information:**

The online version contains supplementary material available at https://doi.org/10.1265/ehpm.24-00279.

## Background

Low birth weight (LBW) of <2,500 g is an important index that reflects the neonatal health status, intra-uterine environment, and maternal nutritional status during pregnancy. LBW-related prenatal malnutrition leads to the risks of early death, developmental retardation [1–3], type 2 diabetes mellitus in adulthood, cardiovascular disease, hypertension [4–6], and psychiatric disease [7, 8], being an important public health issue that affects development and health over a long period. During the WHO’s 65th World Health Assembly convened in Geneva, Switzerland, in 2012, one of 6 goals to be achieved before 2025 was set as “a 30% reduction in the annual incidence of low-birth weight” [9]. However, the currently estimated progress status on a decrease in the prevalence of LBW is slower than that necessary for achieving this goal [10]. Factors for LBW include multiple pregnancy, first childbirth, girls, smoking during pregnancy, a low body mass index (BMI) before pregnancy, insufficient weight gain during pregnancy, mother’s job during pregnancy, late-in-life pregnancy, infertility treatment, father’s absence during pregnancy, and economic situation [3, 11–16]. However, considering that there is no marked decrease in the prevalence of LBW, it is important to identify preventable factors for LBW to be clarified.

LBW is primarily frequent in low- to middle-income countries, such as South Asia and sub-Saharan Africa [10]. However, even in Japan as an advanced country, the number of LBW infants has increased over ≥40 years since 1975 [13, 17–19]. The rate of LBW in Japan (9.4%) is higher than the mean value in OECD countries (6.5%) [20]. Therefore, new findings of factors for LBW other than poverty-related malnutrition may be obtained by conducting a field survey on a specific area of Japan.

As a potential factor for LBW, we assumed that LBW might be associated with Internet use for many hours during pregnancy. Maternity leave and mobility restrictions during pregnancy result in more time at home and less outdoor activity, which may lead to Internet use for many hours [21]. Most pregnant women collect information on fetal growth, complications during pregnancy, lifestyle during pregnancy, and delivery via the Internet, share their experience or thoughts with other pregnant women, and recognize the Internet as a reliable, useful tool [22–29]. Thus, the merits of Internet use are great for pregnant women. However, some studies indicated that Internet use for many hours resulted in problematic Internet use (PIU) in which poor control of Internet use affects personal relationships, social life, and emotional stability [30, 31]. Therefore, prolonged Internet use during pregnancy may lead to PIU with potential impact on prenatal care.

There have been few articles on Internet use for many hours or PIU in perinatal females, but PIU during or after pregnancy (pregnancy period to 1 week after delivery) accounted for as high as 30% [21]. As factors for LBW, a low BMI before pregnancy [14, 16], insufficient weight gain during pregnancy [13, 17], and a low frequency of consultation in the Department of Obstetrics during pregnancy, that is, insufficient prenatal care [11], have been indicated. However, PIU is associated with a decrease in the amount of a meal, skipping meals, thinness [32, 33], and a reduction in health behavior [32]; therefore, Internet use for many hours during pregnancy may lead to LBW. A previous study indicated that maternal PIU was associated with the infant’s thinness [34]. Considering this, Internet use for many hours during pregnancy may affect fetal growth.

The purpose of this retrospective cohort study was to clarify the relationship between Internet use for many hours during pregnancy and LBW in a specific area of Japan, where the rate of LBW is high.

## Methods

### Study design and data sources

The study design is a retrospective cohort study. The target area was Matsue City, Shimane Prefecture. The subjects were mothers who had submitted the pregnancy notification form in Matsue City between April 2016 and September 2017 and their children. Matsue City, Shimane Prefecture, is a provincial city of which the population is approximately 200,000, and the number of births is approximately 1,600/year. In Japan, all pregnant women are obligated to submit the pregnancy notification form to municipalities where they live based on the Maternal and Child Health Act. Most pregnant women submit it before Week 12 of pregnancy. When the municipal office is notified of pregnancy, a public health nurse interviewed mothers to obtain information on the mother’s age, marital status, family structure, and mother’s lifestyle to utilize it for pregnancy/delivery/childrearing support. Furthermore, in Japan, residents are obligated to submit a birth certificate to municipalities where they live within 2 weeks after the newborn’s birth based on the Family Registration Law. During the procedure, local governments obtain information on the neonatal status at the time of birth, such as the birth weight described in the maternal and child health handbook at an obstetric facility. In this study, personally identifiable code-given data were obtained from Matsue City. Matsue City provided the data on Internet use and information on family structure, pregnant women’s age, gravidity, employment status, and presence or absence of smoking, which were entered by pregnant women at the time of pregnancy notification, in addition to the neonatal data on sex and birth weight obtained on birth notification, deleting the individual-identifying name/address/district name/birth date. Before data provision, an outline of this study was published on the websites of Shimane University and Matsue City, and an opportunity for the study subjects to refuse data utilization was established. With respect to ethical considerations, the protocol of this study was approved by the ethics review board for Medical Research Ethics Committee, Shimane University Faculty of Medicine (Approval No. 5923).

**Fig. 1:**
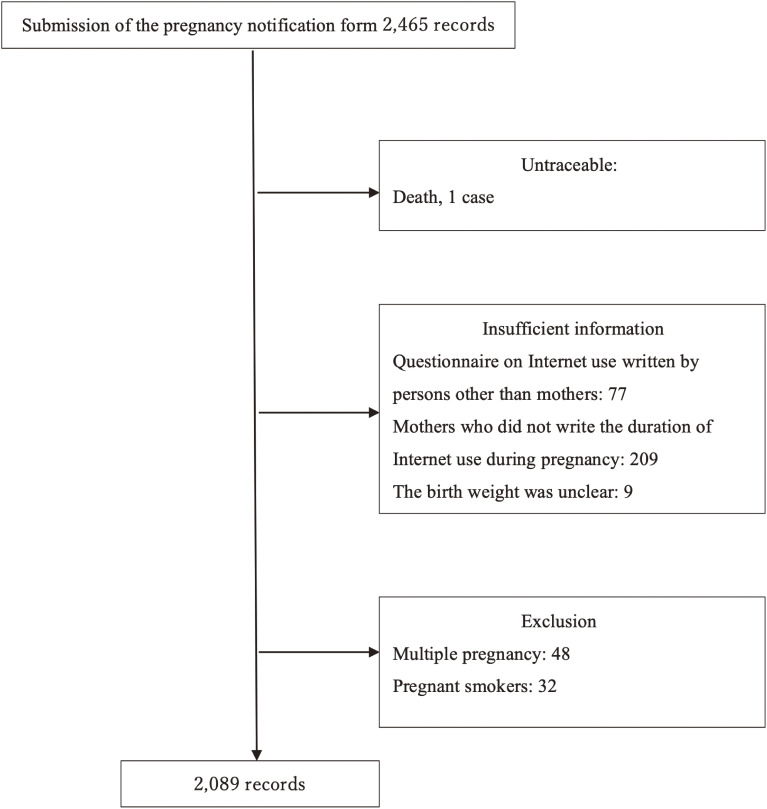
Flowchart of participant selection process

The data provided consisted of 2,465 records. Of these, 1 became untraceable due to death. We excluded 77 records in which the duration of Internet use/day was written by persons other than the mothers on the questionnaire on the notification, 209 in which it was not described, and 9 in which the birth weight was unclear. In addition, we excluded 48 records on multiple birth and 32 records on smoking during pregnancy for the following reasons: these are important risk factors for LBW [35, 36], and the number of episodes is small; therefore, the number of LBW episodes in the population is extremely small, and they are intolerable to multivariate analysis. Finally, we analyzed 2,089 records (Fig. 1).

### Measurements

#### 1) LBW

LBW was defined as a birth weight of <2,500 g regardless of gestational age by the World Health Organization (WHO) [37]. In this study, a birth weight of <2,500 g was also regarded as LBW.

#### 2) Internet use for many hours

We defined Internet use extending over 5 hours, which may lead to PIU with potential impact on prenatal care, as long Internet use [31, 38]. The measurement of Internet use was based on the question presented at the time of pregnancy notification: “How many hours per day on average have you used the Internet for in the past 30 days? Internet use indicates the use of a personal computer, cellular phone, smartphone, or tablet, and includes game or mail usage. It does not include Internet use for work.”

#### 3) Covariates

In reference to the above relevant factors for LBW previously indicated [3, 11–16], we used the child's sex (boy, girl), mother’s age at the time of pregnancy (<35, ≥35), marital status on pregnancy (yes, no), gravidity (≥twice, once), mother’s work during pregnancy (no, yes), and weeks of pregnancy on the notification (<week 12 of pregnancy, ≥week 12 of pregnancy) as covariates. In Japan, there is a system for pregnant women to undergo prenatal checkups at the public expense, and tickets for prenatal checkups are delivered on pregnancy notification. The WHO recommended that pregnant women should receive prenatal care before Week 12 of pregnancy [39]. Therefore, pregnancy notification in ≥Week 12 of pregnancy means delayed prenatal care and a small number of prenatal care sessions.

### Statistical analysis

Logistic regression analysis was performed using LBW as a dependent variable and Internet use for many hours during pregnancy as an independent variable. The odds ratio and 95% confidence interval were calculated. Subsequently, as covariates, the child’s sex, mother’s age at the time of pregnancy, marital status on pregnancy, gravidity, mother’s work during pregnancy, and weeks of pregnancy on the notification were input, and multivariate logistic regression analysis was conducted.

IBM SPSS Statistics 27 was used for the analysis, with the significance level set at <5%.

## Results

Internet use for many hours during pregnancy accounted for 4.4%. LBW accounted for 7.2% (Table 1).

**Table 1:** Characteristics of pregnant women and infants

	**N = 2089**	

	**n**	**%**
Birth weight (n = 2089)		
≥2500	1938	92.8
<2500	151	7.2
Duration of Internet use during pregnancy (n = 2089)		
<5 hours/day	1998	95.6
≥5 hours/day	91	4.4
Child’s sex (n = 2089)		
Boy	1065	51.0
Girl	1024	49.0
Maternal Age (n = 2089)		
Others (<35)	1480	70.8
Advanced (≥35)	609	29.2
Marital status on pregnancy (n = 2082)		
Yes	2031	97.2
No	51	2.4
Gravidity (n = 2089)		
≥Twice	1209	57.9
Once	880	42.1
Work during pregnancy (n = 2078)		
No	410	19.6
Yes	1668	79.8
Gestational age on pregnancy notification (n = 2079)		
<Week 12 of pregnancy	1805	86.4
≥Week 12 of pregnancy	274	13.1

The results of univariate logistic regression analysis showed that LBW was associated with Internet use for many hours during pregnancy (odds ratio = 2.03 (95%CI, 1.08–3.82)) (Table 2).

**Table 2:** Odds ratio of low birth weight and mothers’ Internet use for many hours during pregnancy

		**Crude**		**Adjusted**	

		**OR** **(95%CI)**	**P**	**OR** **(95%CI)**	**P**
Internet use for many hours during pregnancy	Internet use: <5 hours/day	ref		ref	
	Internet use: ≥5 hours/day	2.03 (1.08–3.82)	0.028	2.16 (1.13–4.13)	0.019
Child’s sex	Boy	ref		ref	
	Girl	1.15 (0.83–1.61)	0.400	1.16 (0.83–1.63)	0.381
Maternal Age	<35	ref		ref	
	≥35	1.57 (1.11–2.21)	0.010	1.75 (1.22–2.50)	0.002
Marital status on pregnancy	Yes	ref		ref	
	No	1.75 (0.73–4.16)	0.208	1.75 (0.72–4.23)	0.214
Gravidity	≥Twice	ref		ref	
	Once	1.14 (0.81–1.59)	0.453	1.23 (0.86–1.75)	0.250
Work during pregnancy	No	ref		ref	
	Yes	0.95 (0.63–1.43)	0.798	0.94 (0.62–1.43)	0.784
Weeks of pregnancy on the notification	<Week 12 of pregnancy	ref		ref	
	≥Week 12 of pregnancy	0.95 (0.58–1.57)	0.847	0.97 (0.58–1.60)	0.897

Notes: OR: odds ratio; CI: confidence interval; ref: reference

Adjusted for the child’s sex, maternal age, marital status on pregnancy, gravidity, work during pregnancy, and weeks of pregnancy on the notification

The results of multivariate logistic regression analysis also showed the association between LBW and Internet use for many hours during pregnancy (adjusted odds ratio = 2.16 (95%CI, 1.13–4.13)). Their relationship remained.

There was no multicollinearity of the input variables. Furthermore, the goodness of model fit was examined using the Hosmer-Lemeshow testing for goodness of fit, and the p-value was ≥0.05.

## Discussion

This study clarified the association between Internet use for many hours during pregnancy and birth at an LBW. The results of multivariate logistic regression analysis adjusted with LBW-associated covariates suggested that the risk of LBW under Internet use for many hours during pregnancy is 2.16 times higher than in the absence of such use. The time for pregnant women to spend on obtaining knowledge about delivery via the Internet is reportedly <1 hour per day [23]. Internet use for ≥5 hours may include use for purposes other than collection of information in addition to much time spent on collecting information on delivery, suggesting a potential PIU situation that may lead to inadequate prenatal care.

When considering the mechanisms behind the association between long Internet use during pregnancy and LBW, in the case of PIU, healthy behavior is neglected [32], and prenatal care may be insufficient as much as pregnant women used the Internet. Insufficient prenatal care [11] is a risk factor for LBW; therefore, inadequate prenatal care related to Internet use for many hours during pregnancy may influence birth at an LBW. Furthermore, poor weight gain or thinness, energy-dense, nutrient-poor dietary patterns, imbalances in diet, and a variety of nutritional deficiency during pregnancy [13, 14, 16, 17, 40–42] are risk factors for LBW. In the case of PIU and long Internet use, devotion to the Internet may lead to thinness related to a decrease in dietary intake or skipping meals, nutritional imbalances such as excessive consumption of bread, sweets, sugary drinks, and fast food, coupled with insufficient intake of fruits, vegetables, dairy products, and meat [32, 33, 43–45]; therefore, insufficient nutrient intake related to Internet use for many hours during pregnancy may have contributed to LBW. However, in this study, we did not investigate changes in the nutritional status or body weight during pregnancy. Further studies are required to clarify the association and underlying mechanisms between long Internet use during pregnancy and LBW.

Although our study indicated an association between long Internet use during pregnancy and LBW, it remains unclear whether this association is direct or indirect. Nevertheless, even if the association is indirect, Internet use for many hours during pregnancy may serve as a potential parameter when screening pregnant women at risk of LBW. Therefore, we consider it is necessary to provide appropriate support to pregnant women who use the Internet for many hours during pregnancy after confirming the presence or absence of risk factors for LBW, such as malnutrition, and insufficient prenatal care.

This study has the following 3 limitations: Firstly, a data sample from a provincial city was used, and the sample does not represent Japan; there may be a sample bias. In the future, a similar survey should also be conducted in urban areas, and the number of samples must be increased. However, in this study, the rate of LBW was 7.2%, being within the range from the mean value in OECD countries (6.5%) to that in Japan (9.4%) [20]. The possibility that there may have been a bias requiring special consideration may be low. Secondly, the duration of Internet use per day was based on mothers’ self-reporting, and there may have been an information bias. However, a sense of value to regard Internet use as a bad thing has not been disseminated, and we cannot conclude that there was a unidirectional bias. Thirdly, we could not use factors for LBW, that is, mothers’ thinness, weight gain during pregnancy, smoking and alcohol intake during pregnancy, or economic situations, as covariates. Furthermore, birth defects are a risk factor for LBW [46], but there is no system in Japan to comprehensively track birth defects at birth, which prevented us from excluding cases with birth defects from our analysis. The inability to examine medical factors related to LBW using medical data is a major limitation of this study. In the future, a follow-up survey with these variables, including medical data, should be conducted.

## Conclusions

This study suggested that Internet use for many hours during pregnancy leads to LBW through a longitudinal survey, which is valuable, considering that few studies have examined Internet use for many hours during pregnancy or PIU. In the future, evidence on the relationship between Internet use for many hours during pregnancy and LBW/children’s growth/development must be accumulated by establishing a study design to overcome the above limitations.

## Abbreviations

LBW: Low birth weight
BMI: body mass index
PIU: problematic Internet use

## Supplementary information

**Additional file 1:** **Supplementary Table 1.** The number of occurrences of each independent variable according to LBW status.

## Declarations

### Availability of data and materials

The datasets analyzed during the current study are not publicly available as they are provided and owned by Matsue City, Shimane Prefecture; however, they are available from the corresponding author on reasonable request.
