# Supplementary material for: Association between long Internet use during pregnancy and low birth weight: a retrospective cohort study
Source: Environ Health Prev Med. 2024 Dec 12;29:72. doi: 10.1265/ehpm.24-00279 (PMC11652968; doi:10.1265/ehpm.24-00279)
Supplement: Supplementary file 1 — Additional file 1: Supplementary Table 1. The number of occurrences of each independent variable according to LBW status. [file ehpm-29-072-s001.docx]

*Supplementary information for*

**Association between long Internet use during pregnancy and low birth weight: a retrospective cohort study**

Aya Sakakihara^1*^, Chiyori Haga^2^, Aya Kinjo^3^, Yoneatsu Osaki^3^

^1^ Department of Community Health Nursing, Faculty of Medicine, Shimane University,

89-1 Enya-cho, Izumo City, Shimane Prefecture 693-8501, Japan

^2^ Graduate School of Medicine, Department of Community Nursing, Kagawa University, Kagawa University,1750-1 Ikenobe, Miki-cho, Kita-gun, Kagawa Prefecture 761-0793, Japan

^3^ Division of Environmental and Preventive Medicine, Faculty of Medicine, Tottori University

86 Nishi-cho, Yonago City, Tottori Prefecture 683-8503, Japan

**Correspondence author:**

Aya Sakakihara

Department of Community Health Nursing, Faculty of Medicine, Shimane University, 89-1 Enya-cho, Izumo city, Shimane prefecture, 693-8501, Japan

Email: aya@med.shimane-u.ac.jp

Tel: +81-853-20-2337

Supplementary Table 1. The number of occurrences of each independent variable according to LBW status

|  | Birth weight (N=2089) | | | |
| --- | --- | --- | --- | --- |
|  | ≥2500 | | <2500 | |
|  | *N* | % | *N* | % |
| Duration of Internet use during pregnancy (n=2089) | (n = 1,938) |  | (n = 151) |  |
| <5 hours/day | 1,859 | 95.9 | 139 | 92.1 |
| ≥5 hours/day | 79 | 4.1 | 12 | 7.9 |
| Child’s sex (n=2089) | (*n* = 1,938) |  | (*n* = 151) |  |
| Boy | 993 | 51.2 | 72 | 47.7 |
| Girl | 945 | 48.8 | 79 | 52.3 |
| Maternal Age (n=2089) | (*n* = 1,938) |  | (*n* = 151) |  |
| Others (<35) | 1,387 | 71.6 | 93 | 61.6 |
| Advanced (≥35) | 551 | 28.4 | 58 | 38.4 |
| Marital status at the time of pregnancy (n=2082) | (*n* = 1,932) |  | (*n* = 150) |  |
| Husband’s presence | 1,887 | 97.7 | 144 | 96.0 |
| Husband’s absence | 45 | 2.3 | 6 | 4.0 |
| Gravidity (n=2089) | (*n* = 1,938) |  | (*n* = 151) |  |
| ≥ twice | 1,126 | 58.1 | 83 | 55.0 |
| Once | 812 | 41.9 | 68 | 45.0 |
| Work during pregnancy (n=2078) | (*n* = 1,927) |  | (*n* = 151) |  |
| Absent | 379 | 19.7 | 31 | 20.5 |
| Present | 1,548 | 80.3 | 120 | 79.5 |
| Gestational age on pregnancy notification (n=2079) | (n = 1,929) |  | (n = 150) |  |
| < Week 12 of pregnancy | 1,674 | 86.8 | 131 | 87.3 |
| ≥ Week 12 of pregnancy | 255 | 13.2 | 19 | 12.7 |
